# *Euglena gracilis* paramylon activates human lymphocytes by upregulating pro‐inflammatory factors

**DOI:** 10.1002/fsn3.383

**Published:** 2016-05-20

**Authors:** Rossella Russo, Laura Barsanti, Valter Evangelista, Anna M. Frassanito, Vincenzo Longo, Laura Pucci, Giuseppe Penno, Paolo Gualtieri

**Affiliations:** ^1^ Istituto di Biologia e Biotecnologia Agraria, CNRPisaItaly; ^2^ Istituto di Biofisica, CNRPisaItaly; ^3^ Dipartimento di Medicina Clinica e SperimentaleSezione Malattie MetabolicheUniversità di PisaPisaItaly

**Keywords:** *Euglena*, glucans, innate immunity, MacroGard^®^, paramylon

## Abstract

The aim of this study was to verify the activation details and products of human lymphomonocytes, stimulated by different *β*‐glucans, that is *Euglena* paramylon, MacroGard^®^, and lipopolysaccharide. We investigated the gene expression of inflammation‐related cytokines and mediators, transactivation of relevant transcription factors, and phagocytosis role in cell‐glucan interactions, by means of RT‐PCR, immunocytochemistry, and colorimetric assay. Our results show that sonicated and alkalized paramylon upregulates pro‐inflammatory factors (NO, TNF‐*α*, IL‐6, and COX‐2) in lymphomonocytes. A clear demonstration of this upregulation is the increased transactivation of NF‐kB visualized by immunofluorescence microscopy. Phagocytosis assay showed that internalization is not a mandatory step for signaling cascade to be triggered, since immune activity is not present in the lymphomonocytes that have internalized paramylon granules and particulate MacroGard^®^. Moreover, the response of *Euglena β*‐glucan‐activated lymphomonocytes is much greater than that induced by commercially used *β*‐glucans such as MacroGard^®^. Our *in vitro* results indicate that linear fibrous *Euglena β*‐glucan, obtained by sonication and alkaline treatment can act as safe and effective coadjutant of the innate immune system response.

## Introduction

*β*‐glucans are a group of heterogeneous, highly conserved glucose polymers, which are structural components in fungi and algae, bacteria, and plants. Different *β*‐glucans vary in structures, size, branching frequency, structural modifications, conformation, or solubility in water or alkalis, which may influence their physiological functions and biological activity. The common structure of all *β*‐glucan polymers consists of a main chain of *β*‐(1,3)‐ and/or *β*‐(1,4)‐d‐glucopyranosyl unit in nonrepeating but nonrandom order, along with side chains of varying lengths (Barsanti et al. 2011; Synytsya and Novak 2014).

The most important quality of *β*‐glucans and the reason why so much attention has been devoted to them are the physiological effects they show. They belong to the group of non‐self molecules pathogen‐associated molecular patterns (PAMPS), conserved elements of the pathogenic metabolism, which are nonspecifically recognized by germline‐encoded pattern recognition receptors (PRRs) on the cell surface, forming the base of the innate immune system (Takeuchi and Akira 2010; Thompson et al. 2010). As such, they are typical biological response modifiers with pronounced and potent immunomodulating activity, confirmed in many animal experiments, *in vitro* studies and also in several human clinical studies, predominantly focused on the treatment and prevention of infectious, allergic, and oncologic diseases (Chan et al. 2009; Vetvicka 2011; Jesenak et al. 2014).

The mechanism of *β*‐glucan action in the organism is mediated through several receptors, especially Dectin‐1, Toll‐like receptors, complement receptor 3, scavenger receptor, and lactosylceramide (Goodridge et al. 2009; Kankkunen et al. 2010; Baert et al. 2015). The most important is Dectin‐1, which is highly expressed in many immunocompetent cells such as dendritic cells, neutrophils, eosinophils, macrophages, monocytes, several T lymphocytes and, in humans, also in C lymphocytes. The stimulation of Dectin‐1 activates the innate immune response, ROS, and inflammatory cytokine production through the activation of the transcription factors such as nuclear factor kappa‐light‐chain‐enhancer of activated B cell (NF‐kB), and enzymes such as phospholipase C, and mitogen‐activated protein kinases. Dectin‐1 is indicated as preferential receptor for *β*‐1‐3 linked linear glucans (Tsoni and Brown 2008; Noss et al. 2013; Legentil et al. 2015).

Because of their ability to enhance infection defense mechanisms and simultaneously downregulate inflammations, *β*‐glucans are very promising as an alternative to the mainstream use of immunosuppressive drugs to treat inflammatory diseases. They may form part of a prophylaxis and/or therapeutic strategy following exposure to pathogen challenge, to reduce the need for antibiotics in both human and veterinary medicine, either as dietary supplement or pharmaceutical preparation.

Assessment of the beneficial potential of these compounds and investigation of the structure–function relationship and mechanism of action have been often carried on using poorly described, crude *β*‐glucan preparations, leading to confounding results, misconceptions, and controversies regarding biological effects of chemically well‐defined molecules. Though preparation from plant/algae/fungal source can achieve a high degree of purity, minute sample contaminations may result in nonspecific immune‐modulating effects.

Among *β*‐glucans, paramylon from the unicellular alga *Euglena gracilis* can be considered quite a peculiar case. Paramylon is a linear (unbranched) *β*–(1, 3)–glucan polysaccharide polymer with high‐molecular weight (Barsanti et al. 2011). *Euglena* can accumulate large amounts of this reserve polysaccharide as granules in the cytoplasm up to 95% of the cell mass, when grown in the presence of adequate carbon sources under heterotrophic growth conditions (Barsanti et al. 2001; Monfils et al. 2011). X‐ray diffraction and density measurements indicate that paramylon has a very high level of crystallinity in the native state (about 90%) and consists of 100% glucose as indicated by NMR spectrum (Kiss et al. 1988; Barsanti et al. 2011). The crystallinity of paramylon is due to higher order aggregates of microfibrils, measuring 4–10 nm, composed of unbranched triple helices of *β*‐(1,3)‐d‐glucan chains (Deslandes et al. 1980). Its high crystallinity is an advantage, in that, paramylon granules can be isolated at very low cost and in an efficient manner by simply disrupting the cells and purifying the granules by successive washing with low‐concentration detergent (Barsanti et al. 2001). Paramylon has no solubility in water at ambient temperatures, and its molecular weight is estimated to be larger than 500 kDa (degree of polymerization about 3000) (Sonck et al. 2010).

Paramylon effect has been investigated on both humans and animals (Koizumi et al. 1993; Vismara et al. 2004; Sugiyama et al. 2009, 2010; Kankkunen et al. 2010; Watanabe et al. 2013). It has been proven to potentiate the resistance of *Artemia* brine shrimp to stress conditions resulting from poor growth medium quality and daily handling, and to enhance its reproductive success (Vismara et al. 2004). Results by Sugiyama et al. (2009) have demonstrated that orally administered paramylon exhibits protective action on acute hepatic injury induced by CCl_4_ *via* an antioxidative mechanism. These same authors also demonstrated that the oral administration of paramylon inhibits the development of atopic dermatitis‐like symptoms in mice, providing an effective alternative therapy to prednisolone (Sugiyama et al. 2010). Sonck et al. (2010) have tested paramylon on porcine leukocytes and verified its pronounced activation effect on ROS production by neutrophils and monocytes. Kankkunen et al. (2010) have demonstrated that paramylon as well as other large particulate *β*‐(1,3)‐d‐glucans (curdlan, zymosan, glucan from baker's yeast *Saccharomyces cerevisiae*) are sensed by sophisticated cooperating pathways through both membrane‐bound and cytosolic PRRs, resulting in robust activation of IL‐1*β*‐mediated inflammatory response in human primary macrophages.

Another *β*‐glucan whose wide‐ranging effects are well documented and published is MacroGard^®^, a patented commercial product derived from baker's yeast *S. cerevisiae* cell wall described by the manufacturer (KS Biotec‐Mackzymal, Tromso, Norway) as a feed‐grade *β*‐(1,3),(1,6)–glucan (Baert et al. 2015). It is a highly branched *β*‐(1,3),(1,6)‐glucan having a DP of 400–1500, and is used as non‐medicated additives for livestock and domestic animal feed. This glucan is the most widely documented *β*‐(1,3),(1,6)–glucan for animal feed, and the overall leader as immune stimulant on the fish feed market. It is used as special ingredient in fish feed for salmonids and for marine fish and shellfish (Sych et al. 2013; Kühlwein et al. 2014; Navarrete and Tovar‐Ramírez 2014).

In this article, we investigated the molecular mechanisms leading to activation of peripheral blood mononuclear cell (PBMC) by *β*‐glucans and we delineated their participation in innate immune responses. We analyzed the gene expression of inflammation‐related cytokines and mediators, transactivation of relevant transcription factors, and phagocytosis role in cell‐glucan interactions. The response of *Euglena β*‐glucan‐activated lymphomonocytes with respect to that induced by MacroGard^®^ indicate that fibrous *Euglena β*‐glucan can act as coadjutant factor of the innate immune system response.

## Materials and Methods

### Isolation of peripheral blood mononuclear cells

Venous blood from healthy volunteers was drawn in EDTA (Ethylenediaminetetraacetic acid) tubes and processed within 2 h from collection. PBMC were isolated by low‐speed density gradient centrifugation over Histopaque^®^‐1077 (Sigma Aldrich, density = 1.077 g/mL). PBMC (1 × 10^6^cells/cm^2^) were suspended in RPMI 1640 FBS (Fetal Bovine Serum) 10%, then seeded on multiwell plates or Petri dishes and incubated at 37°C, 5% CO_2._

### Paramylon preparation

Paramylon was extracted and purified from 2‐day‐old *E. gracilis* WZSL mutant according to Barsanti et al. (2001) by suspending a pellet of cells in a solution containing 1% (*w*/*v*) SDS and 5% (*w*/*v*) Na_2_EDTA. The suspension was incubated for 30 min at 37°C and the paramylon granules were recovered by centrifugation at 1000*g* for 10 min. The SDS‐Na_2_EDTA treatment was repeated and the paramylon was washed twice with hot‐glass distillated water (70°C). After the second wash, the granules were deposited on glass fiber filters (APFC type, Millipore Co. KGaA, Darmstadt, Germany), dried overnight at 90°C, and stored at room temperature. MacroGard^®^ (Biotec ASA, Tromso, Norway) was hydrated in deionized water (1.5% *w*/*v*) overnight at 4°C, then collected by centrifugation at 1000*g* for 10 min, and dried overnight under vacuum at room temperature.

### Alkaline treatment of paramylon and MacroGard^®^

Paramylon granules were suspended in NaOH 0.5 mol/L (1% *w*/*v*) and stirred until the solution appeared homogeneous, that is granules were no more detectable by microscopic observation. Glucan was precipitated by adding two volumes of cold 98% ethanol and recovered by centrifugation at 12000*g* for 10 min at 4°C. The pellet was suspended in 40 mL deionized water; ethanol precipitation was repeated, as well as the centrifugation. The final pellet was suspended in 30 mL deionized water. The pH was adjusted to 7.0 with 2 mol/L HCl. A quantity of 1 mL aliquots were prepared and stored at −20°C. Assuming a 20% loss during the procedure, the final concentration of glucan was about 0.8% *w*/*v*. MacroGard^®^ was treated in the same way.

For the tests, both the paramylon and the MacroGard^®^ suspensions were sonicated on ice for 12 min (twelve 48‐sec cycles of sonication with a 12‐sec pause between cycles).

Endotoxin concentration of the two glucans was not tested, since according to Baert et al. (2015), the endotoxin concentration present in paramylon and MacroGard^®^ preparation is consistently lower than 0.5 endotoxin units/*μ*g, which is the FDA limit for sterile water.

### Assessment of cell viability

To screen for the best conditions, toxicity curves with paramylon granules, sonicated and alkalized paramylon, and sonicated and alkalized MacroGard^®^ at different concentration (10 *μ*g/mL, 50 *μ*g/mL, 100 *μ*g/mL, 150 *μ*g/mL, 200 *μ*g/mL, 250 *μ*g/mL, 300 *μ*g/mL) were measured by MTT (3‐(4,5‐dimethylthiazol‐2‐yl)‐2,5‐diphenyltetrazolium bromide). Viability of lipopolysaccharide (LPS) treated cells (10 ng/mL, 50 ng/mL, 100 ng/mL, 500 ng/mL, 750 ng/mL) was measured by trypan blue test.

MTT measures mitochondrial activity in living cells, while trypan blue enter only dead cells. After 3 days of culture, PBMC were incubated with glucans and LPS. The viability assay was carried at 4 and 24 h with 95% viability. Glucans‐treated PBMC were incubated with MTT (Sigma, St. Louis, MO) (1 mg/mL) for 3 h at 37°C, 5% CO_2_. The medium was then removed and the cells solubilized in 10% DMSO/90% Isopropanol. The amount of dye released from the cells was quantified by measuring the optical density at 540 nm with a multiplate reader (ETI‐System Fast Reader, Sorin Biomedica, Sorin‐Livanova PLC, Milan, Italy), since the optical density is directly correlated with the amount of metabolically active cells. Cell viability was expressed as % versus control. LPS‐treated PBMC were washed with PBS and detached by means of trypsin‐EDTA. Cells were collected by centrifugation (5 min, 1000*g*), suspended in growth medium, and mixed 1:1 with trypan blue (Sigma). Viable cells were counted in a Burker chamber. All treatments were performed in triplicates.

The concentration resulting from the dose–response curves were 150 *μ*g/mL for the glucans and 500 ng/mL for LPS (data not shown).

### Immunocytochemistry of NF‐kB

Translocation of NF‐kB to the nucleus was evaluated by fluorescence microscopy. Briefly, after 24 h treatment, PBMC were washed in PBS and fixed with −20°C methanol for 20 min. Then, cells were washed, incubated with 0.1% Triton X‐100 for 10 min, blocked with 1% BSA for 1 h, and incubated with rabbit polyclonal anti‐NF‐kB p65 (phosphor S536, Abcam, Cambridge, UK) as primary antibody and Alexa Fluor 647‐donkey anti‐rabbit IgG (H+L) antibody (Life Technologies, Carlsbad, CA) as secondary antibody at room temperature. The slides were extensively washed with PBS and viewed with a fluorescence microscope. DAPI was used as nuclei‐specific dye.

### Quantitative real‐time PCR

Gene expression of interleukin‐6 (IL‐6), cyclooxygenase 2 (COX‐2), tumor necrosis factor alpha (TNF‐*α*), and inducible nitric oxide synthase (iNOS) was quantified with real‐time PCR and *β*‐actin was used as housekeeping gene. After 24 h treatment with 150 *μ*g/mL glucan or 500 ng/mL LPS, total RNA was isolated from PBMC using the RNeasy Mini Kit (Qiagen, Hilden, Germany) and reverse‐transcribed by the iScript^TM^ cDNA Synthesis Kit (Bio‐Rad, Hercules, California, CA). Quantitative real‐time PCR was performed using the SoFastTM EvaGreen^®^ Supermix (Bio‐Rad, Hercules, California, CA) in the StepOnePlus^TM^ Real‐Time PCR System (ABI Applied Biosystems, Foster City, CA). Gene primers (Table 1) were designed using Beacon Designer Software (Premier Biosoft, Paolo Alto, California, USA). Samples were assayed in triplicate and the gene expression was calculated using the 2^−ΔΔCt^ relative quantification method. The amount of various transcripts was normalized to *β*‐actin transcript in the same cDNA sample. Results were expressed as fold‐increase with respect to control and plotted as mean ± SD of three independent experiments.

**Table 1:** Primers sequence used for quantitative Real‐Time PCR

Primers	Forward	Reverse
*β*‐actin	5′‐GAGATGCGT‐TGTTACAGGAAG‐3′	5′‐TGGACTTGGGAGAGGACT
TNF*α*	5′‐AACCCTCAGACGCCACAT‐3′	5′‐CGGATCATGCTTTCAGTGC‐3′
COX‐2	5′‐CCGAGGTGTATGTATGAGTGT‐3′	5′‐CTGTGTTTGGAGTGGGTTTC‐3′
IL‐6	5′‐AAAGCAGCAAAGAGGCAC‐3′	5′‐TTCACCAGGCAAGTCTCC‐3′
iNOS	5′‐CTCAAGGACAGGTCTCT‐3′	5′‐GTCACTTATGTCACTTATCTGGAT‐3′

### Nitrite (NO^−^_2_) determination

Nitrite level was used as indicator of nitric oxide (NO) production and was quantified in culture media by Griess reaction, according to the manufacturer's protocol (Sigma Aldrich, Saint Louis, Missouri, USA). The optical density was read at 540 nm using a microplate reader (ETI‐System Fast Reader, Sorin Biomedica). Nitrite concentration was expressed as % versus control using a nitrate standard curve (0–500 *μ*mol/L). This assay was performed on cellular supernatant after 4 and 24 h treatment with 150 *μ*g/mL of glucans and 500 ng/mL of LPS on 3‐day‐old PBMC. Samples were assayed in triplicate and results were plotted as mean ± SD of three independent experiments.

### Light and fluorescence microscopy

The hardware platform used to acquire transmitted and fluorescence images consists of a Zeiss Axioplan microscope (Zeiss, Oberkochen, Germany), equipped with an epifluorescence system, a 100× (N.A. 1.30) planapochromatic objective, and a 100 W stabilized tungsten‐filament lamp. Fluorescence images were acquired with a digital color CCD camera (Basler scA160028 fm/fc, Basler AG, Ahrensburg, Germany) equipped with a IEEE 1394b interface mounted in the TV microscope path. The resolution of the original image is 1628 × 1236 pixels.

### Photography

Transmission photographs were recorded with an Olympus Camedia C‐3030 digital camera (Olympus, Shinjuku, Tokyo, Japan) mounted on a Zeiss Axioplan microscope (Zeiss, Germany).

### Scanning electron microscopy (SEM) preparations

For SEM observation, *β*‐glucans were pipetted onto a poly‐l‐lysine coated coverslip. After dehydration in graded acetone solutions, samples were dried in a critical‐point dryer apparatus, coated with gold and viewed using a Philips‐SEM 505 microscope (Eindhoven, The Netherlands).

### Statistical analysis

The statistical analysis was performed using GraphPad Prism, version 6.00 (GraphPad Software, La Jolla, CA). Assays were carried out in triplicate and results were expressed as mean ± SD of three independent experiments. Differences between samples were analyzed by one‐way analysis of variance. Differences were considered significant when *P* < 0.01.

## Results

**Figure 1:**
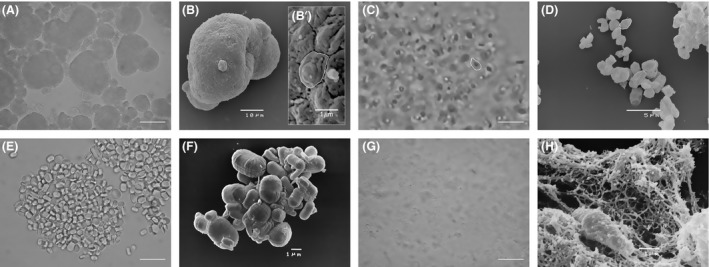
Overall aspect of MacroGard^®^ and paramylon: (A) optical microscopy image of MacroGard^®^ aggregates dispersed in distilled water; 20 *μ*m bar; (B) SEM image of a single aggregate; (B’) magnification of the aggregate surface showing its particulate texture; the white contour defines a single particle; (C) homogeneous particulate mixture of sonicated and alkalized MacroGard^®^; the white contour defines a single particle; 5 *μ*m bar; (D) SEM image of the particles present in the mixture; the white contour defines a single particle; (E) optical microscopy image of paramylon granules dispersed in distilled water; 5 *μ*m bar; (F) SEM images of the granules; (G) heterogeneous mixture of sonicated and alkalized paramylon; 10 *μ*m bar; (H) SEM image of paramylon sonicated and alkalized showing its linear fibrous structure.

The glucans used in our experiments are shown in Figure 1. The overall aspect of the raw materials is quite different; examination of MacroGard^®^ powder dispersed in distilled water revealed aggregates ranging from 5 to 50 *μ*m in diameter as clearly shown in Figure 1A and B. After sonication and alkaline treatment, the material appeared as a homogeneous mixture, consisting of individual 1–2 *μ*m particles (Fig. 1C and D) composing the aggregates (Fig. 1B’). A single particle is shown framed in Figure 1B’, C, and D. Examination of paramylon revealed that the raw polysaccharide after isolation from *Euglena* biomass consisted of typically large and small ellipsoid granules less than twice as long as wide, ranging from 1 to 2 *μ*m (Fig. 1E and F). After sonication and alkaline treatment, the granules lost their crystalline nature and appeared as heterogeneous material (Fig. 1G) with a net‐like fibrous structure (Fig. 1H). It is worthwhile to emphasize that the scanning microscopy preparation caused the aggregation of the processed materials and these artifacts are especially visible in Figure 1D, F, and H.

**Figure 2:**
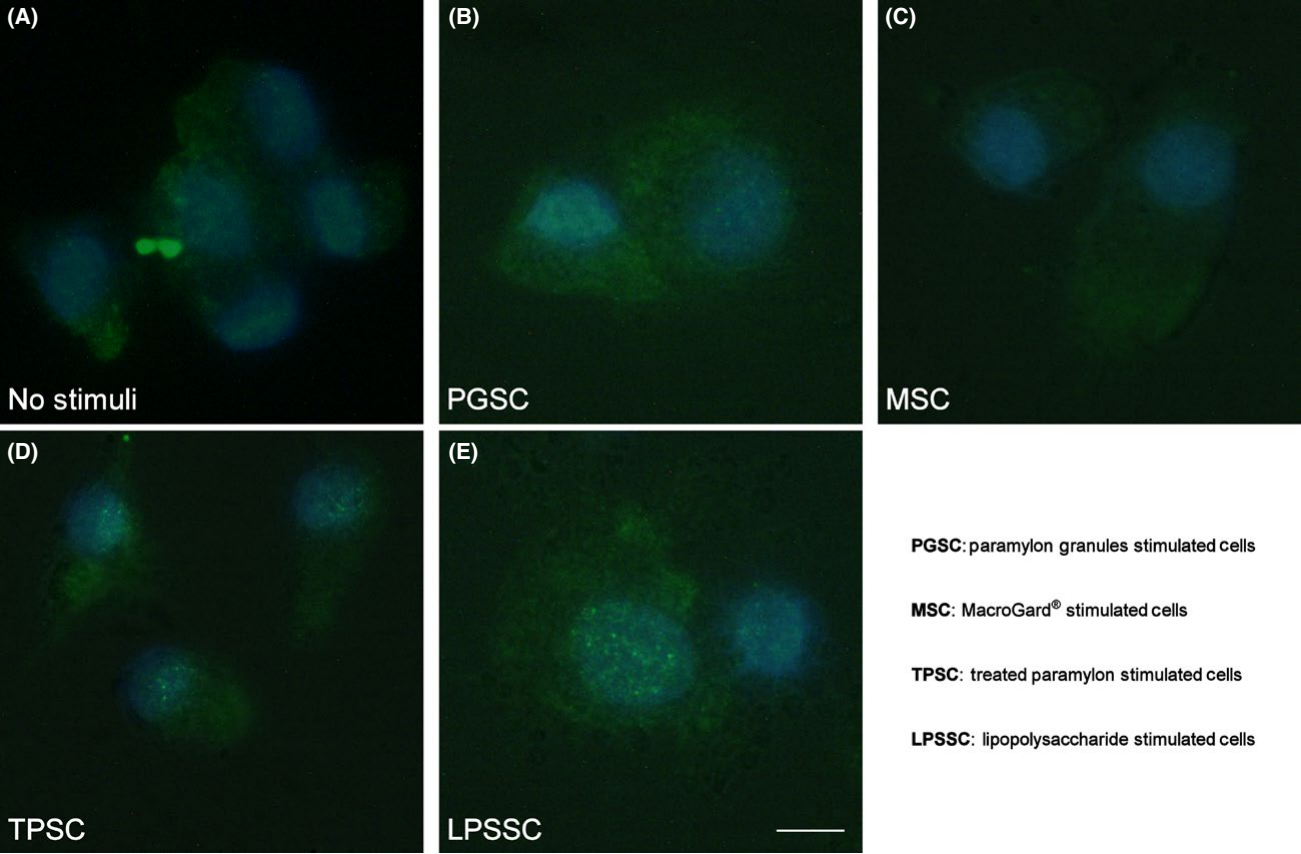
Translocation of nuclear factor nuclear factor kappa light‐chain‐enhancer of activated B cell visualized by fluorescence microscopy: (A) nonstimulated peripheral blood mononuclear cell (PBMC); (B) paramylon granules stimulated PBMC; (C) MacroGard^®^ stimulated PBMC; (D) sonicated and alkalized paramylon stimulated PBMC; (E) lipopolysaccharide stimulated PBMC. Bar 10 *μ*m.

Figure 2 shows the translocation of nuclear factor NF‐kB to the PBMC nucleus evaluated by fluorescence microscopy. The NF‐kB p65 antibody does not label the nuclei of nonstimulated cells, paramylon granules stimulated cell (PGSC), and MacroGard^®^ stimulated cell (MSC) (Fig. 2A, B, and C). A clear labeling is present in treated paramylon stimulated cell (TPSC), which is however lower than the strong labeling shown by lipopolysaccharide‐stimulated cell (LPSSC) nuclei (Fig. 2D and F).

**Figure 3:**
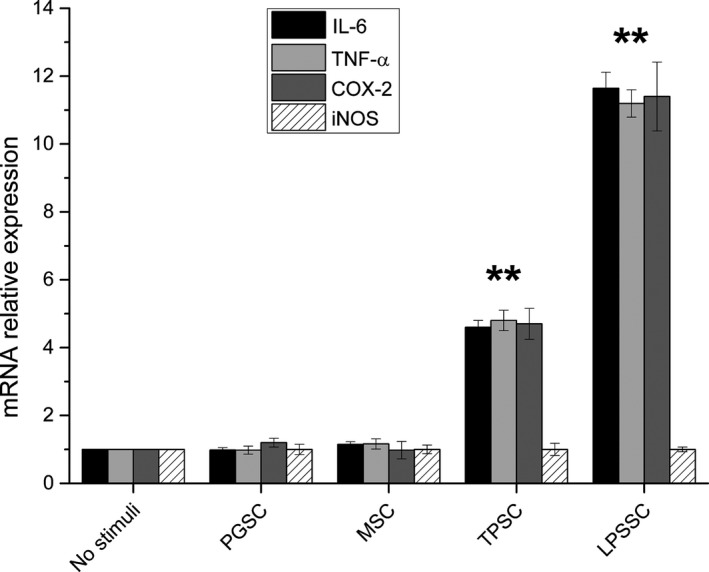
Relative expression of interleukin‐6, tumor necrosis factor alpha, cyclooxygenase 2, and inducible nitric oxide synthase in peripheral blood mononuclear cell after 24 h stimulation with glucans and lipopolysaccharide. Results were expressed as fold‐increase respect to control and plotted as the mean ± SD. ** = *P* < 0.01 versus no stimuli.

Figure 3 represents the relative expression of pro‐inflammatory mediators (IL‐6, TNF‐*α*, COX‐2, and iNOS) after 24 h stimulation of PBMC with glucans and LPS. TPSC expressed a higher level (*P* < 0.01 vs. no stimuli) of the two cytokines and COX‐2 with respect to PGSC and MSC, whose expression level is not statistically different from that of nonstimulated cells. LPS stimulated cells, which are used as positive control, express very high levels of IL‐6, TNF‐*α* and COX‐2 (*P* < 0.01 vs. no stimuli). After 24 h, the stimulated cells express an iNOS level comparable with that of nonstimulated cells.

**Figure 4:**
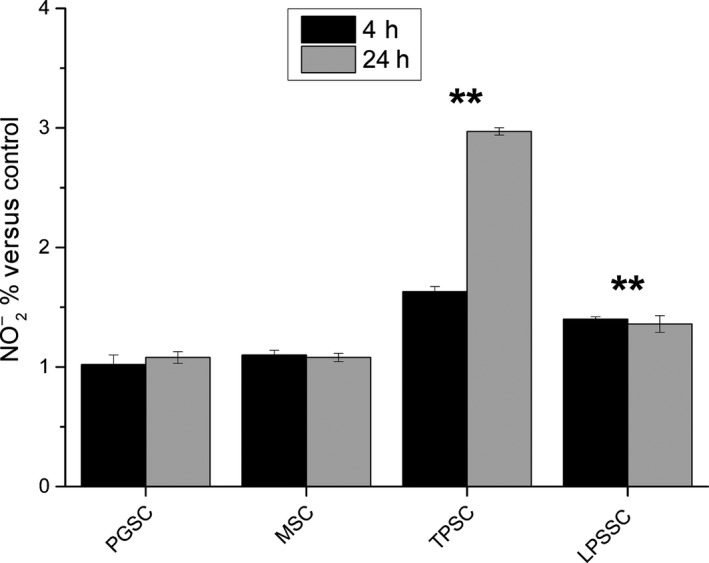
Nitric oxide production by peripheral blood mononuclear cell stimulated with glucans and lipopolysaccharide after 4 and 24 h, expressed as percent nitrite concentration versus control. ** = *P* < 0.01 versus no stimuli.

PBMC culture medium was assessed for nitrite levels to evaluate NO production after 4 and 24 h treatment with 150 *μ*g/mL of glucans and 500 ng/mL LPS. Figure 4 shows the time course of NO production by stimulated PBMC. After 4 h, the amount of NO produced by TPSC is about 1.5 times (about 46 *μ*mol/L, *P* < 0.01), higher than that produced by nonstimulated cells (about 31 *μ*mol/L). After 24 h, there is a further increase up to three times the production by nonstimulated cells (about 97 *μ*mol/L, *P* < 0.01). After 4 h, the amount of NO produced by LPSSC is about 1.4 times higher than that produced by nonstimulated cells, and this amount does not show any further increase after 24 h (about 43 *μ*mol/L, *P* < 0.01). The amount of NO produced by PGSC and MSC is statistically not significantly different from that produced by nonstimulated cells after 4 and 24 h.

**Figure 5:**
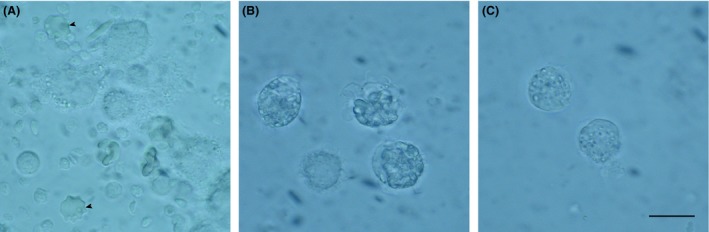
Results of the phagocytic assay: (A) Peripheral blood mononuclear cell (PBMC) surrounded by sonicated and alkalized paramylon and dendritic cells typical of immune activation (arrowheads); (B) paramylon granules visible inside PBMC; (C) MacroGard^®^ particles visible inside PBMC. Bar 10 *μ*m.

Figure 5 shows the result of phagocytic assay performed on PBMC incubated with the different glucans. Sonicated and alkalized paramylon is visible surrounding the cells and monocyte‐derived dendritic cells typical of the immune activation are present in the microscope field (arrow heads, Fig. 5A); paramylon granules and Macrogard^®^ have been phagocytized by PBMC, but monocyte‐derived dendritic cells are absent in both samples (Fig. 5B and C).

## Discussion

Three different stimuli, that is treated MacroGard^®^, paramylon granules, and treated paramylon granules, were chosen to test the influence of source, primary chemical structure, and three‐dimensional conformation on the activation of cellular innate immunity reactions. LPS was used as a positive control, since it is a potent and prototypical inducer of cytokine production in innate immunity (Alexander and Rietschel 2001).

The glucans used in our experiment derived from two different sources, and have different primary structure and different three‐dimensional conformation.

MacroGard^®^ is a commercial preparation derived from the cell wall of the yeast *S. cerevisiae*. This glucan is the most widely documented glucan for animal feed. The primary chemical structure consists mainly of branch‐on‐branch *β* 1,3/1,6 glucan, with a reported purity of about 60% (Alaban et al. 2014). MacroGard^®^ consists of aggregates of about 50 *μ*m (Fig. 1A), which can be reasonably interpreted as a patchwork of irreducible particles resulting from the heterogeneous mixture of the component molecules (Fig. 1B and B’). As demonstrated by Tabata and Ikada (1988), PBMC do not interact in any form with particle greater than 1–2 *μ*m; hence, aggregates were sonicated and alkalized to obtain smaller particles. Sonication produces 1–2 *μ*m size particles, while alkaline treatment does not produce any further chemical or structural modification (Fig. 1C and D). Our images of MacroGard^®^, before and after treatments, are comparable to those of Hunter et al. (2002), who showed a preparation obtained from common baker's yeast using alkalization and acidification procedures. According to these authors, the irreducible particles contain hexoses, proteins, lipids, and nucleic acid impurities.

Paramylon is the reserve polysaccharide of *E. gracilis* WZSL mutant (Rosati et al. 1996). The primary chemical structure of paramylon consists exclusively of a linear chain of *β*‐(1,3) glucopyranosyl units, with a very high level of crystallinity in the native state and no contaminants as demonstrated by the NMR spectrum (Barsanti et al. 2011). This glucan is stored inside the cells as small ellipsoid granules ranging from 1 *μ*m to 3 *μ*m (Fig. 1E and F). Sonication does not produce any fracture, while alkaline treatment does reduce the degree of polymerization of original paramylon from about 3000 to about 70, and the molecular weight from about 500 kDa to about 12 kDa (Tamura et al. 2009). After this treatment, paramylon appears as a heterogeneous moiety made up of fibrous structures consisting of linear chain(s) of *β*‐(1,3) glucopyranosyl units, visible in Figure 1G and H. In the latter figure, aggregation and thickening of the fibers are due to the SEM preparation procedure.

Paramylon, MacroGard^®^, and LPS are PAMPs and play a pivotal role in the initiation of a variety of anti‐pathogen defense mechanisms, by interacting with a wide variety of cell surface receptors (e.g., PRR). Such actions lead to systemic inflammatory response, via upregulation of pro‐ and anti‐inflammatory cytokine genes, which results in secretion of cytokine into the blood stream (Mogensen 2009; Takeuchi and Akira 2010; Newton and Dixit 2012).

We followed the immune response of PBMC upon stimulation by different glucans and LPS, from the activation of transcription factor to the induction of pro‐inflammatory mediators. We monitored the transactivation of NF‐kB (“rapid acting” primary transcription factor), a first responder transcription factor to harmful cellular stimuli (Nishikori 2005). In its inactive form, NF‐kB is present in the cytoplasm bound by its inhibitor IkB; upon stimulation, IkB is degraded by IkB kinase and NF‐kB is free to move to the nucleus, where it can bind to response elements of its target genes. As a consequence, pro‐inflammatory mediator expression is induced, and mediator is produced (Kobayashi 2010). We evaluated the expression of four important pro‐inflammatory mediators of antibody responses along different pathways such as IL‐6 (pro‐inflammatory cytokine), TNF‐*α* (cell‐signaling cytokine), COX‐2 (enzyme for synthesis of prostaglandins), and iNOS (enzyme for synthesis of NO) (Janský et al. 2003; Schulte et al. 2013; Ali et al. 2015). Then, we assessed NO production by stimulated PBMC, since iNOS‐derived NO is an important cellular signaling molecule secreted as free radical (Lechner et al. 2005).

Our results show that sonicated and alkalized paramylon has an immune‐activating effect similar to LPS. In fact, both stimuli increased transactivation of NF‐kB as visualized by immunofluorescence microscopy (Fig. 2), while MacroGard^®^ and paramylon granules do not produce any labeling. As a consequence, upregulation of pro‐inflammatory factors (TNF‐*α*, IL‐6, and COX‐2) is present only in TPSC and LPSSC, which show the highest amount of expression (Fig. 3). Despite the low level of iNOS gene expression found in TPSC and LPSSC after 24 h of stimulation, which is not statistically different from the expression by nonstimulated cells, MSC and PGSC (Fig. 3), the amount of iNOS‐induced NO produced by TPSC and LPSSC was much higher than that produced by the other groups of cells and increased from 4 h to 24 h (Fig. 4), with TPSC producing more NO than LPSSC. This last finding may be due to the kinetic of iNOS expression that probably reaches its maximum level and decays within 24 h and is higher in TPSC respect to LPSSC. This auto‐inhibitory effect via transcription inhibition of iNOS gene by NO per se was already reported (Chang et al. 2004). The high level of NO produced by TPSC exerted a similar inhibitor effect on pro‐inflammatory cytokines expression via inhibition of NF‐kB transactivation as shown by the lower amount of labeling respect to LPSSC (Fig. 2D and E) (Katsuyama et al. 1998). On the contrary, the lower level of NO produced by LPSST was not sufficient to exert an inhibitory action on cytokine expression, whose high level in turn increased NF‐kB transactivation by a positive feedback mechanism. This chronic activation could lead to a dangerous cytokines storm (Tisoncik et al. 2012). In the case of TPSC, the lower level of cytokines expression indicates that there is no further induction of NF‐kB activation, thus assigning this paramylon preparation a positive role of safe immune‐stimulating agent.

Finally, phagocytosis assay showed that internalization is not a mandatory step for signaling cascade to be triggered, since immune activation, marked by the presence of dendritic cells (Fig. 5A, arrowheads), is not present in the lymphomonocytes that have internalized paramylon granules and particulate MacroGard^®^ (Fig. 5B and C).

We can conclude that the immune‐stimulating activity of glucans necessitates of the recognition and linkage with a membrane receptor, which in our experiment is limited only to the sonicated and alkalized paramylon, due to its linear fibrous structure. Both paramylon granules and MacroGard^®^ lack the ability to link to the membrane receptors due to their particulate structure. Diverse studies have indicated that linear glucans interact preferentially with Dectin‐1 receptor, and that this interaction becomes tighter as the length of the glucan chain increases (Hanashima et al. 2014; Donadei et al. 2015). These data can explain the effect of the sonicated and alkalized paramylon with respect to both paramylon granules and MacroGard^®^ on PBMC, though further studies will be needed to clarify the binding of fibrous paramylon to membrane receptors. These *in vitro* results indicate that *Euglena* paramylon, sonicated and alkalized, can act as a safe, effective coadjutant factor of the innate immune system response.

## Conflict of Interest

None declared.
